# Are pre-school girls more likely to be under-nourished in rural Thatta, Pakistan?-a cross-sectional study

**DOI:** 10.1186/s12939-015-0287-3

**Published:** 2015-12-21

**Authors:** Rozina Nuruddin, Wilbur C. Hadden

**Affiliations:** Department of Community Health Sciences, Aga Khan University, Stadium Road, P.O. Box 3500, Karachi, 74800 Pakistan

**Keywords:** Preschool children, Gender, Under-nutrition, Pakistan, WHO growth reference

## Abstract

**Background:**

Pakistan ranks third lowest on a global gender index (2013) and 13^th^ highest on the prevalence of underweight among under-five children (2010). Through this population-based study, we examined gender differentials in the prevalence of stunting, wasting and under-weight defined by World Health Organization (WHO) Growth Standard among rural pre-school Pakistani children.

**Methods:**

We performed secondary analysis of data collected through a cross-sectional survey of Thatta district during 1992-93. Prevalence ratios were calculated for 1051 children aged 0-35 months from 95 randomly selected villages of rural Pakistan using a clustered adjusted log binomial model. Level 1 variables included child and household characteristics and level 2 included village characteristics.

**Results:**

Based on the new WHO growth reference, a major proportion of children were stunted (52.9 %), wasted (22.9 %) and under-weight (46.5 %). In a two-level model, compared to boys, girls had significantly greater risk of stunting [Prevalence Ratio (PR) (95 % C.I.) = 1.18 (1.03, 1.36)] and under-weight [P.R. (95 % C.I.) 1.14 (1.03, 1.26)], after adjustment of maternal literacy and village variables. Risk of wasting did not differ with gender [P.R. (95 % C.I.) = 1.04 (0.99, 1.15)]. Mothers of stunted and underweight children were respectively, 21 and 20 % more likely to be illiterate than those of normally nourished children. Sick children were at 16 % greater risk of wasting than those not reported ill.

**Conclusion:**

Greater prevalence of stunting and under-weight among girls suggests adoption of a gender sensitive approach in nutritional intervention programmes. Prompt management of childhood illnesses may reduce prevalence of wasting. Better literacy among rural mothers may reduce prevalence of stunting and under-weight. Whether gender differences in nutrition status are an underlying pathway for excessive girl mortality in rural Thatta needs further examination.

## Background

Under nutrition is a major modifiable determinant of child survival, health [1, 2], growth and development [3]. However, it continues to be a major public health problem in most developing countries. More than half of the world’s undernourished children are found in just three countries (India (39 %), Bangladesh (5.7 %) and Pakistan (5.5 %) [4]. Pakistan ranks 57 out of 76 countries on the global hunger index (based on population under nourishment, child underweight and child mortality) [5]. Its recent estimates of underweight, wasting and stunting for under-five Pakistani children are 31.6, 10.5 and 45 %, respectively [6]. Further, these estimates are higher for rural (33.3, 16.1 and 46.3 %) than for urban population (26.6, 12.7 and 36.9 %) [7]. Of concern is worsening status of stunting between 1990–99 estimates (42.7 %) [8] and 2012–13 estimates (45 %) [6].

Gender differences in nutritional status are of concern mainly in settings where girls are considered less important than boys [9]. On a global gender index (based on four fundamental categories of economic participation and opportunity, educational attainment, health and survival and political empowerment), Pakistan ranking is dropped from third lowest out of 135 countries in 2011 [10] to second lowest out of 142 countries in 2014 [11]. Evidence about gender difference in undernutrition among Pakistani children however, is scant and conflicting. Girl disadvantge in nutritional status is reported in eight urban squatter settlements of Karachi [12], boy disadvantage in 300 rural and urban communities included in the Pakistan Integrated Household Survey (PIHS) (1991) [13] and no gender difference in four rural districts of Sindh [14]. These findings did not account for contextual community level factors in analysis. In addition, they were based on nutritional status of children defined by 1977 NCHS growth reference, the suitability of which as a growth reference has been criticized on serious technical grounds [15, 16]. The new WHO growth reference released in April 2006 is recommended for use in preference to the NCHS reference to assess children regardless of ethnicity, socio-economic status and type of feeding [17]. It gives greater prevalence of stunting throughout childhood, of wasting during infancy and of under-weight during first half of infancy [18, 19]. Pakistan is reported to use combined NCHS/WHO growth reference, combined growth charts for males and females and z-score or standard deviation system rather than percentiles [20]. However, gender differential in nutritional status of pre-school rural Pakistani children has not been assessed based on new World Health Organization (WHO) growth reference.

**Fig. 1:**
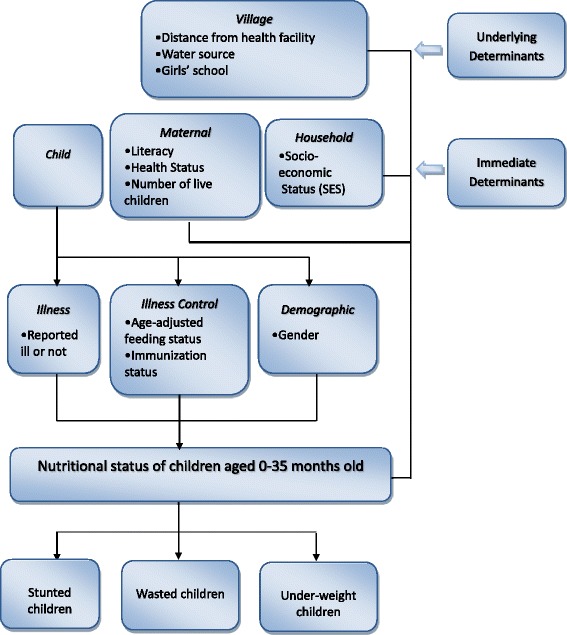
Conceptual framework of association between child’s nutritional status and gender

The conceptual frameworks proposed by Mosley and Chen [21] and by UNICEF [22] describe gender as a determinant of dietary intake (a proximate determinant) based on cultural and societal values. The UNICEF framework [21] identifies inadequate dietary intake and disease as immediate causes of under-nutrition, food security, care of women and children, health services and environment as underlying causes, and economic and political structures as basic determinants of under-nutrition. In this study, we grouped our variables into two instead of three levels of UNICEF framework as follows; (i) *underlying or intermediate level determinants* that relate to the community and consist of village factors and (ii) *immediate or proximal level determinants* that relate to the individual and consist of child, maternal and household characteristics (Fig. 1).

We primarily aimed to examine the association of gender separately with stunting (reflecting chronic under-nutrition), wasting (reflecting acute under-nutrition) and under-weight (reflecting synthesis of acute and chronic under-nutrition) among pre-school rural Pakistani children in specific conceptual and analytical frameworks. Through a two-level logistic regression model, we tested the null hypothesis that child nutritional status was not influenced by gender.

## Methods

### Study setting, design, data source and participants

Thatta is a predominantly rural district of Sindh province located 60 km east of Karachi inhabited by a predominantly Muslim and Sindhi speaking population [23, 24] of about 1.1 million [25]. Its growth rate is 2.26 with an average household size of 5.1 and dependancy ratio of 87.9 [25]. With an under five mortality rate of 129/1000 live births and with 49 % of its under-five children underweight, its health indicators are the worst of 16 districts of the Sindh province [26]. Its developmental indicators are also poor such as 35 % adult (≥15 years) literacy level [27] and access to piped water for only 16–17 % of the popualtion [26, 27]. The death rate among girls is also relatively higher than among boys during postnatal period and in the 12–59 months age group [28]. The adult sex ratio in Thatta (112 M :100 F) is greatly baised towards males [29].

The data analysed for this cross-sectional study are derived from a larger survey conducted by the Aga Khan University, Karachi during November 1992 and February 1993 in Thatta, a rural district of Sindh Province of Pakistan. This survey provided baseline information on heath and nutrition status of the population and use of government health services to be used in evaluating a health system improvement project [23, 24]. Information was collected at the village, household and individual levels and provided relevant measures of our conceptual framework. The more recent Pakistan Demographic Health Survey (2012–13) lacks information about village development such as access to health facilities and availability of girls’ schools, maternal health status and age-adjusted feeding status of children [30]. Moreover, our data though collected more than two decades ago are still relevant as Thatta has not shown significant improvement in health and developmental indicators over time. According to UNICEF, Thatta was categorized as a district with low Human Development Index [26]. Its child health indicators have worsened over time as shown by under-five mortality rate /1000 live births of 108 in 1992–93 [31] and 129 in 2003-04 [26], infant mortality rate/1000 live births of 78 in 1992–93 [31] and 91 in 2003–04 [26] and underweight prevalence of 48 % for children under three in 1992–93 [18] and of 49 % for children under five in 2003–04 [26]. Its progress on development indicators is also not impressive such as an adult (>15 years) literacy level of 32 % in 1992–93 [23] and 35 % in 2012–13 [27] and concrete housing for 17 % of population in 1992–93 [31] and 19 % in 2004–05 [32].

A three-staged stratified random cluster sampling technique was adopted. First, union councils (UCs) were identified that served as strata. Second, villages were identified that served as clusters or sampling units. Third, households within selected villages were surveyed to provide information about population elements. From a total of 24 Union Councils (UC), 12 with fairly complete enumeration lists were identified. Due to eccentric location of some of the government health facilities (GHF) within a UC, a 5 km radius around them was drawn. Villages (Primary Sampling Units) located within this radius were listed and mapped. From the available list of villages, through computer-generated random numbers, five to twelve villages were selected from each service area, with a target to sample at least 250 households per GHF catchment area. Only households having at least one child less than five years of age were surveyed. In this way, a population of 24,121 subjects from 2,276 households and ninety-nine villages were surveyed, giving an average of 23 households per village. Non-response rate was 9 % for overall survey and 30 % for anthropometric examination of children aged 0–35 months. In this way, from 952 households and 95 villages, 1051 children participated in anthropometric examination and were included in the study [18]. This was more than desired sample of 922 children based on assumption of an annual crude birth rate of 36/1000 population and a 40 % prevalence of under-weight among children aged 0–35 months [24], an α error of 5 %, a desired precision of 5 % and a design effect of 2.5 [33].

### Data collection

An elaborate pre-coded survey questionnaire designed for a face-to-face interview was pre-tested after approval from an institutional ethical committee. A validation survey of 400 households was conducted within two weeks of the actual survey for determination of data collection errors [24]. Informed consent was obtained from village headmen and heads of hosueholds to provide information about relevant sections of the questionnaire. Mothers were approached to provide active constent regarding child anthropometric examniation.

Village headmen provided information for a village profile including water source and presence of girls’ school. Village distance by the shortest possible road route to the nearest health facility was measured (in kilometres) using a vehicle odometer.

Heads of households provided information about three indicators of household socio-economic status (SES) (i.e. type of house, land ownership and per capita average monthly household income, later divided at its median) which were combined to create a single measure. Subjects were grouped as low, middle or upper SES if all three, any two or at most one of three indicators reflected economic disadvantage.

Mothers were asked about their literacy status (ability to read and/or write a short simple statement) and number of live children. Their health status during the year preceding the interview was assessed through a series of questions about illness symptoms lasting for > 2 weeks. They also provided information about their children. This included age determined with the aid of a calendar listing important local events, festivals and moon cycles in the last five years, any illness during the last one year, age at weaning and immunization status (assessed by immunization card when available or from mother).

Trained field workers took anthropometric measurements of children under three years of age. Childrens’ weight was recorded to the nearest 0.1 kg using portable 25 kg spring balance Salter Scales (Salter England, West Bromwich, UK). Weighing scales were calibrated daily using 20 kg weights. Standard weighing procedures were followed. Children were lightly clad and without shoes/slippers. Recumbent length (for children less than 24 months) was measured to the nearest centimetre with portable flat wooden boards with sliding foot pieces (locally manufactured by Pakistan Medical and Dental Council). For children older than 24 months, standing height was obtained. Severely malnourished children were referred to a local hospital or a health centre for further assessment and care.

Completed questionnaires were checked and validated by field supervisors daily. Questionnaires with inconsistencies were re-sent to the field for correction. Data quality was maintained by supervision and retraining of the field staff.

Categorization of the study variables is listed in table 1.

**Table 1:** Description of study variables and their categorization

Independent variables	Categories^a^
Child characteristics	
Gender	Girl or boy
Age-adjusted feeding status	• Aged ≥ 12 months and on regular diet • Aged 7–11 months and weaned at or after 7 months • Aged 5–11 months and weaned at 5–6 months • Nursing infants aged 0–6 months
Immunization status	Incomplete/none or complete/ appropriate.
Reported ill during one year period	Yes or no
Maternal and household characteristics	
Maternal literacy	Illiterate or literate
Number of live children	≥4 or ≤ 3
Poor maternal health	Yes or no
Household socio-economic status (SES) (House type, land ownership, income)	Low, middle or upper
Village characteristics	
Distance from the nearest health facility	>3 or ≤ 3 km
Water source	Non-piped (well, pond, canal or river) or piped
A girls’ school.	Absent or present

^a^ Last category is the reference category for all variables

### Data analysis

Measured heights and weights were converted to standard normal scores (Z-scores) on the WHO standard distributions adjusting for child’s age and gender with a software package named ANTHRO (available at http://www.who.int/nutgrowthdb). Our Z-score standard deviations were close to 1, suggesting reasonable quality of the measures [34].

To account for unequal selection probabilities and to reduce bias in variance estimation, weights were calculated as the inverse of the sample selection probabilities. We present weighted prevalence of under-nutrition with their 95 % confidence intervals. Prevalence of under-nutrition (percentage of children aged 0–35 months) was calculated following convention [35] as the number of children with Z-scores less than 2 SD below the WHO standard for nutritional parameters wasting (weight-for-height), stunting (height-for-age) and underweight (weight-for-age). Mild, moderate and severe malnutrition were determined using the relevant parameters of the reference population as below -1 and down to −2 SD, below −2 and down to −3 SD and below −3 SD, respectively. Mean Z-scores (and their SD) for under-nutrition were calculated to compare with the WHO standard.

Since this study was a part of the larger survey, subjects who met the criteria for this study were identified from the database and the post-hoc power calculations were done assuming an α error of 0.05, a design effect of 2 and 20 % greater under-nutrition among girls than among boys. The post hoc power for detecting gender difference in prevalence of stunting, wasting and under-weight, respectively was found to be 74, 90 and 94 %. As under-nutrition is a relatively common outcome measure (with a prevalence of >10 %), we calculated adjusted prevalence ratios [36] using SAS Proc Genmod [37] with the binomial distribution and the log-link function [38]. We adopted COPY method when the log-binomial model did not converge [39].

Clustering at the village level was accounted for by the use of a cluster identitfier for village level variables in a repeated statement using Proc Genmod. We did not account for clustering at household or maternal level as there was only one child less than 35 months per household and per mother in 90 and 92 % respectively of the surveyed households. Hence, our level-1 (micro-level) variables were child, maternal and household factors and level-2 (macro-level) variables were village factors.

Five multivariate models were estimated for each nutritional status variable as follows: (i) gender and village variables; (ii) village variables only; (iii) child and village variables; (iv) gender, household and village variables; and (v) gender and village variables along with those child and household variables identified in crude and model 3 or 4 analyses. In the final model, gender and village variables were retained and the variable with the smallest *p*-value (<0.05) was entered first, followed by the addition of one variable at a time, retaining ones with *p*-value ≤ 0.05 and removing ones with *p*-value > 0.05. We present the results of generalized estimating equations from the final model.

## Results and discussion

### Characteristics of the study population

There were fewer girls than boys. Nursing infants (0-6 months) constituted 21.3 % of the subjects. Based on the new WHO growth reference, a significant proportion of children were stunted (52.9 %), wasted (22.9 %) and under-weight (46.5 %) (Table 2). More than half had not received any immunization and about a quarter received incomplete immunization as reported by mothers or documented in the immunization card ( 38.8 % of children). About one fifth (18.0 %) of the children were reported ill over a one year period.

**Table 2:** Child characteristics (*n* = 1051)

Variables	Per cent (weighted)
Stunted children	52.9
Wasted children	22.9
Under-weight children	46.5
Female	47.0
Age-adjusted feeding status • Nursing infants (0–6 months of age) • Infants aged 5–11 months and weaned at 5–6 months • Infants aged 7–11 months and weaned at or after 7 months • Early childhood ( ≥12 months of age)	21.3 11.0 10.0 57.7
None or incomplete immunized children	79.7
Children reported ill	18.0

Most of the mothers were illiterate (83.7 %) and had four or more living children (52.5 %). One-fifth of them (21.9 %) reported poor health. Households with low, middle and high SES consitituted 27.6, 48.8 and 23.6 % of the population, respectively. Most villages were three or more kilometres away from the nearest government health facility (55.4 %), had a non-piped water source (89.9 %) and no girls’ school (82.9 %).

### Risk factors for under-nutrition (un-adjusted analysis)

In univariate analyses, risk of stunting was greater for girls (by 19 %) and for children of illiterate mothers (by 25 %) (Table 3). Wasting was not significantly associated with gender. However, its risk was 17 % greater among children reported sick. Risk of under-weight was greater for girls (by 13 %), if mother was illiterate (by 25 %) and if water source was non-piped (by 20 %).

**Table 3:** Crude prevalence ratios for under-nutrition (confidence intervals adjusted for clustered design)

Variables	Crude prevalence ratios (95 % C.I.)		
	Stunting	Wasting	Under-weight
Child characteristics			
Female	1.19 (1.03, 1.37)	1.05 (0.99, 1.12)	1.13 (1.02, 1.27)
Early childhood ^a^	0.89 (0.76, 1.04)	1.04 (0.95, 1.13)	1.07 (0.89, 1.27)
Infants weaned late ^a^	0.88 ( 0.69, 1.11)	0.98 (0.88, 1.09)	0.97 ( 0.72, 1.31)
Infants weaned early ^a^	0.99 (0.82, 1.20)	0.93 (0.81, 1.06)	1.02 (0.79, 1.30)
None/incomplete immunization	1.01 (0.84, 1.22)	0.96 (0.87, 1.07)	0.88 (0.76, 1.02)
Reported ill	0.95 (0.79, 1.15)	1.17 (1.05, 1.31)	0.83 (0.65, 1.06)
Maternal and Household characteristics			
Illiterate mother	1.25 (1.03, 1.52)	0.99 (0.91, 1.07)	1.25 (1.06, 1.47)
≥4 live children	0.98 (0.85, 1.14)	1.01 (0.94, 1.09)	1.01 (0.88, 1.16)
Poor maternal health	0.98 (0.83, 1.15)	1.05 (0.97, 1.13)	1.04 (0.91, 1.18)
Households of low SES ^b^	1.05 (0.89, 1.24)	1.04 (0.93, 1.14)	0.95 (0.78, 1.14)
Households of middle SES ^b^	1.03 (0.87, 1.23)	1.07 (0.98, 1.17)	1.10 (0.94, 1.29)
Village characteristics			
Health facility at ≥ 3 km	0.86 (0.71, 1.03)	1.01 (0.94, 1.09)	0.87 (0.74, 1.03)
Non-piped water supply	0.84 (0.67, 1.06)	0.93 (0.86, 1.01)	1.20 (1.01, 1.43)
No girls’ school	0.96 (0.75, 1.22)	0.99 (0.90, 1.09)	0.99 (0.79, 1.23)

^a^ Reference category is nursing infants

^b^ Reference category is households of high SES

### Gender and under-nutrition (a two-level analysis)

Compared to boys, girls had 18 and 14 % greater risk of stunting and under-weight, respectively, after adjustment of maternal literacy and village variables (Table 4). Similarly, children with illiterate mothers were at 21 and 20 % greater risk of being stunted and under-weight respectively than literate mothers after accounting for child’s gender and village variables.

**Table 4:** Under nutrition and gender: adjusted prevalence ratios (95 % CI)

Variables	Adjusted prevalence ratio (95 % C.I.)		
	Stunting	Wasting	Underweight
Child characteristics			
Female	1.18 (1.03, 1.36)	1.04 (0.99, 1.15)	1.14 (1.03, 1.26)
Early childhood ^a^	–	–	–
Infants weaned early ^a^	–	–	–
Infants weaned late ^a^	–	–	–
None/incomplete immunization	–	–	–
Reported ill	–	1.16 (1.04, 1.30)	–
Maternal and household characteristics			
Illiterate mother	1.21 (1.01, 1.46)	–	1.20 (1.02, 1.41)
≥4 live children	–	–	–
Poor maternal health	–	–	–
Low SES ^b^	–	–	–
Middle SES ^b^	–	–	–
Village characteristics			
Health facility at ≥ 3 km	0.91 (0.77, 1.07)	1.04 (0.96, 1.11)	0.93 (0.79, 1.09)
Non-piped water supply	1.14 (0.92, 1.41)	1.07 (0.98, 1.16)	1.15 (0.98, 1.35)
No girls’ school	0.97 (0.79, 1.18)	0.98 (0.90, 1.07)	1.02 (0.86, 1.21)
Log Likelihood	−680.73	−530.34	−679.70
R-Square ^c^	0.009	0.004	0.008

^a^ Reference category is nursing infants

^b^ Reference category is households of high SES

^c^ Max-Rescaled Generalized R-Square

Gender was not a significant predictor of wasting when adjustment was made for child illness and village variables. However, sick children were at 16 % greater risk of wasting than those not reported ill after accounting for child’s gender and village variables.

All contextual factors had a null effect on stunting, wasting and under-weight. Overall, village variables contributed little to explain variability in child’s nutritional status.

### Discussion

Based on Millennium Development Goal 1 (MDG1) (halving the prevalence of underweight from 40 % in 1990 to <20 % in 2015 among under-five children), Pakistan’s progress (31.5 % in 2013) is off the track [40]. Compared to other provinces that are showing declining trends, there is negligible progress on MDG1 for Sindh province where Thatta district is located [40].

This study supports the existence of girl disadvantage in child nutrition in Thatta district. Higher prevalence of stunting (by 18 %) and under-weight (by 14 %) among girls than boys suggests examining a possible role of under-nutrition as an underlying pathway for excessive girl mortality. These findings are in contrast to the recent reports of Pakistan Demographic and Health Survey (PDHS) (2012–2013) [30] which shows greater prevalence of stunting, wasting and under-weight among boys than among girls under 5 years of age. Though, PDHS uses WHO growth standard as the reference, these national figures do not simultaneously adjust for gender and other variables including age.

However, our findings are consistent with those observed in Bangladesh [41] where WHO growth reference was used to study the gender difference in height for age and where girls showed greater growth faltering than boys after 23 months of age. Association of gender with stunting observed in our study is weaker compared to that reported by Baig et al [12] for urban squatter settlements of Karachi. This could be because of the use of a different growth reference [42]. Irrespective of gender, use of WHO growth reference has been reported to detect stunting better among children and to detect underweight and wasting better among young infants [18, 19]. In reality, the prevalence of under-nutrition among girls could be more than that observed since anthropometric measurements were done only for surviving children, leaving better nourished girls to be measured and excluding those who died of severe under-nutrition [43].

This study provides additional evidence associating child’s illness and maternal illiteracy with under-nutrition. Increased risk of wasting with child’s illness suggests association of acute under-nutrition with severe infections [44]. Maternal literacy may influence stunting and under-weight through better management of household resources, better weaning and feeding practices and improved health knowledge and behaviour [45, 46].

Although water source showed an association with wasting and under-weight in one of the preliminary models, it lost its significance later in the final model. This could in part be due to the fact that access to improved water source does not, on its own, ensure improved health. It is its correct use through hygienic behaviour such as washing hands with soap after defecating and before eating and safe handling while preparing food and storage of water that leads to improved health [47, 48].

Evidence regarding a gender differential in nutritional status in South Asia is scant compared to evidence regarding a gender differential in health care seeking. Most of the available evidence does not support the existence of female disadvantage in nutritional status, despite strong son preference in South Asia [14, 49, 50] except for a few studies that suggest female disadvantage in nutritional status [12, 51]. Important determinants of such discrimination against girls include lower socio-economic status [52], older siblings of similar gender [53] and birth order [9]. In all these studies however, the nutritional status of children was not based on the new WHO growth standard, odds ratio was the measure of effect reported and analysis did not account for contextual community factors.

### Strengths and limitations

Our study is unique in that its conclusions are based on the most appropriate measure of association (prevalence ratio) suitable for common outcome measures such as child under-nutrition. Studies based on odds ratios in such circumstances tend to overestimate common outcomes [36]. In this study, we also take account of cluster correlation. Further, nutritional status is defined based on the recent WHO growth standard. Assessment of gender differentials in nutritional status in a fairly comprehensive conceptual framework suggests that a greater proportion of girls are stunted and wasted compared to boys in the rural district of Thatta with significant influence of maternal illiteracy on nutritional status.

Since the survey was conducted during a dry winter period before the harvest season, low access to food might have accounted for high levels of under-nutrition. A certain degree of misclassification could not be excluded particularly for variables based on self-report such as child’s age, household income, maternal health status and child feeding, immunization and health status. There is however, a close relationship between prevalence of stunting and underweight obtained from computed ages and by ages rounded to the nearest month [54]. Non-availability of anthropometric information for 30 % of the subjects is almost similar to the missing values reported for more than 25 % of population in UNICEF, district-based Multiple Indicator Cluster Survey [26]. Though, age, gender, mean household income per capita and maternal education did not differ significantly by response status [18], it is likely that those who did not participate could be ill and under-nourished. Despite low study power (74 %) to detect the gender differential in stunting, we were able to detect it.

Selected UC were those that had enumerated village list reflecting their better administrative capacity compared to those where complete village list was not available. This could have resulted in exclusion of UC with worse health and developmental indicators. Similarly, selection of villages located within 5 kms radius distance from GHF because of convenience could reflect inclusion of better off villages and could affect generalizability of the results. In addition, we were unable to account for other important determinants of nutritional status such as mother’s anthropometry, child’s birth weight and birth order, sibling gender and birth interval due to lack of such information in our dataset.

## Conclusions

Greater prevalence of stunting and under-weight among girls suggests that a gender sensitive approach should be adopted in nutritional intervention programmes. Communities, governmental and non-govenrmental organizations should be sensitized towards the long term and inter-generational consequences of girl malnutrition on delivery complications such as obstructed labour due to short pelvis or birth of small babies. Similarly, children with reported illnesses and whose mothers are illiterate constitute high risk groups for nutritional advice. Prompt management of childhood illnesses may reduce prevalence of wasting and improvement of maternal literacy among rural mothers may reduce prevalence of stunting and under-weight.

Exploration of gender differences in nutrition status should consider follow-up of a birth cohort to address the concerns of age misreporting and survivor bias and to permit inclusion of information about maternal anthropometry, child’s birth weight, birth interval, birth order and sibling gender in the conceptual framework. Use of anthropometric scores in such studies should be based on the new WHO growth reference. Further, determinants of malnutrition at the household level such as the reasons for intra-household discrimination in allocation of food and whether gender differences in nutrition status are an underlying pathway for excessive girl mortality in rural Thatta needs examination.
